# Lecithin-Coated PLGA Nanoparticles for Pulmonary Targeting of Naringin: Formulation, Optimization and In Vitro Characterization

**DOI:** 10.3390/ijms27115095

**Published:** 2026-06-04

**Authors:** Pooja Dattatray Deshmane, Sanjeevani Shekhar Deshkar, Avinash Kharat, Ramesh Bhonde, Ravindra Wavhale, Prabhanjan Giram

**Affiliations:** 1 Dr. D. Y. Patil Unitech Society’s, Dr. D. Y. Patil Institute of Pharmaceutical Sciences and Research, Pune 411018, Maharashtra, India; pdeshmane642@gmail.com (P.D.D.); ravindra.wavhale@dypvp.edu.in (R.W.); 2 School of Pharmacy and Research, Dnyaan Prasad Global University, Pune 411018, Maharashtra, India; 3 Regenerative Medicine Laboratory, Dr. D.Y. Patil Dental College and Hospital, Pune 411018, Maharashtra, India; avinashkharat25@gmail.com (A.K.); rrbhonde@gmail.com (R.B.); 4 Department of Pharmaceutical Sciences, The State University of New York, Buffalo, NY 14214, USA

**Keywords:** naringin, polymer nanoparticles, pulmonary drug delivery, lung targeting, cellular uptake, reactive oxidative stress, in vitro anti-inflammatory activity

## Abstract

Chronic obstructive pulmonary disease (COPD) is a progressive respiratory disorder characterized by persistent airflow limitation and chronic airway inflammation. Current therapeutic strategies primarily offer symptomatic relief and are often limited by systemic side effects, inadequate lung deposition, and poor patient compliance. Naringin (NAR), a natural flavonoid with strong antioxidant, anti-inflammatory, and anti-fibrotic activities, has demonstrated potential in mitigating COPD-associated pathophysiology. However, its therapeutic application is restricted by poor water solubility, low bioavailability, and rapid metabolism. Nanotechnology-based drug delivery systems, particularly poly(lactic-co-glycolic acid) (PLGA) nanoparticles, provide an effective approach for lung-targeted therapy. Their nanoscale size promotes deep lung deposition, enhanced cellular uptake, reduced lung clearance, improved therapeutic efficacy, and reduced systemic side effects. The present study aimed to develop NAR-loaded PLGA nanoparticles (NAR PLGA NP) for enhanced cell-targeting in inflammatory lung conditions. NAR PLGA NP were prepared using the emulsion solvent evaporation method, with PLGA in the organic phase and soya lecithin (SL) with poly(vinyl alcohol) (PVA) as surfactants in the aqueous phase. A face-centered central composite design was employed to optimize the formulation. The optimized nanoparticles were characterized for size distribution by dynamic light scattering, entrapment efficiency, Transmission Electron Microscopy (TEM), Fourier Transform Infrared (FTIR), Differential Scanning Calorimetry (DSC), X-Ray Diffraction (XRD), and in vitro drug release. The safety of PLGA and lecithin-coated PLGA nanoparticles (LC PLGA NP) was assessed using an MTT assay on lung epithelial cells, followed by cellular uptake studies, angiogenesis by chick Yolk Sac Membrane (YSM) assay, and in vitro evaluation of reactive oxidative stress (ROS) and anti-inflammatory activity. The optimized PLGA formulation showed a hydrodynamic diameter of 201 ± 1 nm with PDI 0.20 ± 0.03 and EE of 76.11 ± 2.1%, and 81.7 ± 4.9% drug release at 72 h, whereas LC PLGA NP showed a hydrodynamic diameter of 308 ± 3 nm, PDI of 0.21 ± 0.05, entrapment efficiency of 82.45 ± 4.8%, and 71.4 ± 3.2% drug release at 72 h. Both PLGA NP and LC PLGA NP demonstrated good cytocompatibility with lung epithelial cells, efficient cellular uptake, and a significant reduction in intracellular reactive oxygen species (ROS) levels (**** *p* value < 0.0001). Moreover, the formulations markedly suppressed pro-inflammatory cytokines, including TNF-α, IL-6, and IL-1β, indicating anti-inflammatory activity. The angiogenesis assay further suggested their ability for lung tissue repair and remodeling. These findings support the potential of LC PLGA NP as a promising cell-specific targeting system for naringin in inflammatory lung conditions.

## 1. Introduction

Chronic obstructive pulmonary disease (COPD) has a substantial impact on health and is the third most common cause of death globally [1]. In low-income or underdeveloped countries, COPD cases are common, resulting in poor health and a reduced quality of life. A history of Tuberculosis (TB) infection, biomass, pollution, and tobacco smoke are the main risk factors [2]. Excessive production of free radicals and long-term lung inflammation are the root causes of COPD-mediated airway damage, which results in airway blockage and breathing difficulties [3]. Patients with COPD pathology and cardiovascular problems have an even more complex issue, which leads to poor patient outcomes. Currently, the therapeutic management of COPD primarily includes bronchodilator therapy, smoking cessation, antibiotic treatment during infectious exacerbations, and the use of corticosteroids. Symptomatic relief is commonly achieved through inhaled combination therapies comprising long-acting muscarinic acetylcholine receptor antagonists (LAMAs) and long-acting β_2_-adrenergic receptor agonists (LABAs). However, these pharmacological interventions are largely limited to symptomatic management and do not directly address the underlying inflammatory pathology of COPD [4,5]. Although corticosteroids have demonstrated some clinical benefit, particularly in reducing exacerbation frequency, their anti-inflammatory efficacy remains limited as COPD-associated inflammation progresses and becomes increasingly steroid-resistant.

Herbal medicines offer a promising approach for the management of COPD by targeting crucial pathological mechanisms beyond symptomatic relief. Plant-derived bioactive compounds possess potent anti-inflammatory and antioxidant properties, which suppress chronic airway inflammation and oxidative stress by modulating signaling pathways such as NF-κB and Nrf2 [6]. In addition, certain herbal actives may promote epithelial repair, inhibit apoptosis, and regulate airway remodeling, thereby supporting endogenous tissue repair and limited regenerative processes [7]. Although complete lung regeneration in advanced COPD is unlikely, herbal medication may help slow disease progression and improve lung function by enhancing cellular repair and restoring airway homeostasis [8].

Naringin (NAR) (4′,5,7-trihydroxyflavanone-7-rhamnoglucoside) is a naturally occurring flavanone glycoside predominantly found in citrus fruits. It has attracted considerable research interest due to its antioxidant, anti-inflammatory, anti-apoptotic, and immunomodulatory activities across various disease models [9]. NAR and its aglycone metabolite, naringenin, exert free radical scavenging effects, enhance expression of endogenous antioxidant enzymes (e.g., catalase, superoxide dismutase), and modulate key signaling pathways implicated in oxidative stress and inflammation [10]. In animal models, compounds structurally related to naringin have demonstrated improvement in COPD-like symptoms by suppressing pro-inflammatory cytokines, reducing neutrophil infiltration, and inhibiting NF-κB activation, one of the master regulators of inflammatory responses in COPD [11].

Recent preclinical evidence further supports the potential of NAR in pulmonary inflammation and oxidative-stress-related lung injury [12]. In a chronic obstructive pulmonary disease mouse model induced by combined lipopolysaccharide (LPS) and cigarette smoke, oral administration of naringin significantly ameliorated histopathological lung injury, suppressed inflammatory cell infiltration, decreased pro-inflammatory cytokines, reduced oxidative stress markers (e.g., malondialdehyde), and improved endothelial barrier function by upregulating aquaporin-1 expression [13]. Additionally, naringin has been shown in other lung injury models to attenuate inflammation and oxidative stress by inhibiting MAPK/NF-κB signaling and activating the Nrf2/HO-1 antioxidant pathway, indicating its ability to modulate both pro-inflammatory and antioxidant defense mechanisms [14]. The Nrf2 pathway plays a pivotal role in cellular antioxidant defenses and is dysregulated in COPD, leading to decreased expression of critical antioxidant genes. Activation of Nrf2 has been associated with enhanced expression of antioxidant enzymes and reduced oxidative damage in COPD models, highlighting this pathway as a promising therapeutic target [15,16].

Nanotechnology-based drug delivery systems have shown promise in pulmonary drug delivery by providing targeted and regulated therapeutic activity [17]. Among these systems, polymeric nanoparticles (NP) have attracted significant attention due to their biocompatibility, biodegradability, tunable size and surface properties, and their ability to encapsulate diverse therapeutic payloads ranging from small molecules to nucleic acids [18,19]. Polymeric NPs can penetrate mucus barriers, protect labile drugs from degradation, and facilitate sustained release at the site of disease, thereby enhancing therapeutic efficacy and reducing dosing frequency, features that are particularly advantageous in the context of COPD, where chronic inflammation and oxidative stress are central to pathogenesis [20,21]. Inhalable polymer and lipid–polymer hybrid nanocarriers have demonstrated improved lung targeting and local drug retention, enabling more effective management of inflammatory responses while limiting systemic side effects [22,23]. Polymeric NPs have also been explored for the delivery of microRNA therapeutics to modulate inflammatory gene expression in COPD models, indicating their utility beyond conventional small molecules [24]. Furthermore, advanced polymeric designs, including pH-responsive and surface-modified nanoparticles, can enhance mucus penetration and cell-specific uptake, addressing key physiological barriers in diseased lungs [25].

Among polymeric materials, poly(lactic-co-glycolic acid) (PLGA) stands out due to its approved safety profile, biodegradability into endogenous metabolites (lactic and glycolic acids), and versatility in tailoring drug release profiles and particle characteristics [26,27]. Surface modifications (e.g., lecithin, PEG, or targeting ligands) further expand PLGA therapeutic utility by enhancing stability, mucus penetration, cellular uptake, and lung deposition. These attributes make PLGA-based NP highly promising platforms for delivering anti-inflammatory and antioxidant agents, including natural compounds with poor solubility and limited bioavailability [28].

Surface modification of nanoparticles with lecithin and PEG to enhance hydrophilicity has been extensively explored for stability and tissue retention [29]. Lecithin, a naturally derived phospholipid mixture rich in phosphatidylcholine, has been investigated as a biomimetic coating material. The pulmonary surfactant layer lining the alveoli is primarily composed of phospholipids, particularly dipalmitoyl phosphatidylcholine (DPPC). Lecithin coating provides a biologically compatible interface between nanoparticles and the alveolar microenvironment [30,31]. Lecithin-coated PLGA nanoparticles (LC PLGA NP) combines the structural integrity and uptake properties of PLGA with the surface-active, membrane-compatible characteristics of phospholipids. This hybrid core–shell system enhances cellular uptake, lung targeting, reduces aggregation in pulmonary fluids, and improves interaction with alveolar epithelial cells. Furthermore, the phospholipid shell may facilitate improved mucus penetration, reduced macrophage recognition, and prolonged pulmonary residence time [32,33]. Such properties are particularly advantageous in inflammatory lung conditions like COPD, where epithelial integrity is compromised, and oxidative stress is elevated. Overall, lecithin-coated PLGA nanoparticles represent a rational and innovative approach for lung-targeted drug delivery, providing enhanced cellular uptake, improved deposition, and avoiding rapid lung clearance [34,35].

The present study aimed to develop LC PLGA NP for cell-specific targeting and improving efficacy. The LC PLGA NP were prepared by the emulsion-solvent evaporation technique. In this system, PLGA forms the biodegradable polymeric core, with NAR incorporated as the anti-inflammatory therapeutic agent. Encapsulation of NAR within the PLGA core enables sustained drug release, improved stability, and enhanced pulmonary retention, potentially improving therapeutic efficacy in COPD. Soya Lecithin (SL) was incorporated as a shell material surrounding the PLGA core, forming a lipid–polymeric nanoparticle system. The lecithin shell enhances physicochemical stability, improves biocompatibility, and may facilitate better interaction with the pulmonary epithelial surface. Additionally, the lipid layer can contribute to improving drug entrapment efficiency and cellular uptake, supporting cell-specific targeting.

The nanoparticle system was optimized using a face-centered central composite statistical design, with PLGA: NAR ratio (X_1_) and poly(vinyl alcohol) (PVA) concentration (X_2_) as the independent variables. To assess the formulations’ physicochemical characteristics, hydrodynamic diameter, polydispersity index (PDI), zeta potential, and drug entrapment efficiency (EE) of nanoparticles were measured. The surface morphology of the prepared nanoparticles was examined using transmission electron microscopy (TEM). Physicochemical properties and potential interactions among formulation components were further determined through Fourier transform infrared (FTIR) spectroscopy, differential scanning calorimetry (DSC), and X-ray diffraction (XRD) analyses. In vitro drug release studies were performed to investigate the release profile of the developed formulations. Cytotoxicity of PLGA NP and LC PLGA NP was assessed in lung epithelial cells using the MTT (3-(4,5-dimethylthiazol-2-yl)-2,5-diphenyltetrazolium bromide) assay to determine cell viability. Cellular uptake studies were conducted using confocal laser scanning microscopy (CLSM) and flow cytometry using the A549 lung cell line to evaluate the internalization efficiency of the nanoparticles.

## 2. Result and Discussion

### 2.1. Optimization of Naringin PLGA NP

In this study, we designed PLGA NP and LC PLGA NP containing NAR for pulmonary targeting. The emulsification process was used to develop the LC PLGA NP. SL and PVA, non-ionic surfactants, were used to encapsulate NAR within the hydrophobic PLGA core and form a self-assembling shell around it. PLGA was selected because it is biodegradable, and the hydrophilic layer of soy lecithin would stabilize the NP and facilitate deeper lung tissue penetration. The optimization by central composite design involved different levels of PLGA: NAR ratio (X_1_) and PVA concentration (X_2_) to obtain desirable responses of hydrodynamic diameter (Y_1_), PDI (Y_2_), and EE (Y_3_) (Table 1). The hydrodynamic diameter of the NAR PLGA NP was found to be in the range of 201.2 ± 1.3 to 330.4 ± 10.7 nm. The entrapment efficiency was analyzed by the dialysis bag method and found to be in the range of 57.12 ± 4.2 to 77.23 ± 5.1%.

The ANOVA data (Table 2) show how factors X_1_ and X_2_ influence hydrodynamic diameter, PDI, and %EE. The 2FI model was the best fit for all responses (Y_1_, Y_2_, and Y_3_). The significance of the model is confirmed by the F-values (13.11 for Y_1_, 4.25 for Y_2_, and 4.97 for Y_3_) and *p*-values less than 0.05 (0.017 for Y_1_, 0.045 for Y_2_, and 0.023 for Y_3_).

Equations (1), (2), and (3) depict the effects of PLGA: NAR ratio (X_1_) and PVA concentration (X_2_) on hydrodynamic diameter (Y_1_), PDI (Y_2_), and EE (Y_3_), respectively.

Hydrodynamic diameter (Y_1_) = +214.69 − 26.80X_1_ + 10.80X_2_ + 8.93X_1_X_2_ + 35.99X_1_^2^ + 61.59X_2_^2^ (1)

PDI (Y_2_) = +0.1588 + 0.0490X_1_ + 0.0413X_2_ + 0.0670X_1_X_2_ + 0.1051X_1_^2^ + 0.0361X_2_^2^ (2)

EE (Y_3_) = +74.07 + 3.73X_1_ + 0.5450X_2_^2^ − 0.2250X_1_X_2_ − 4.40X_1_^2^ − 8.63X_2_^2^ (3)

**Figure 1:**
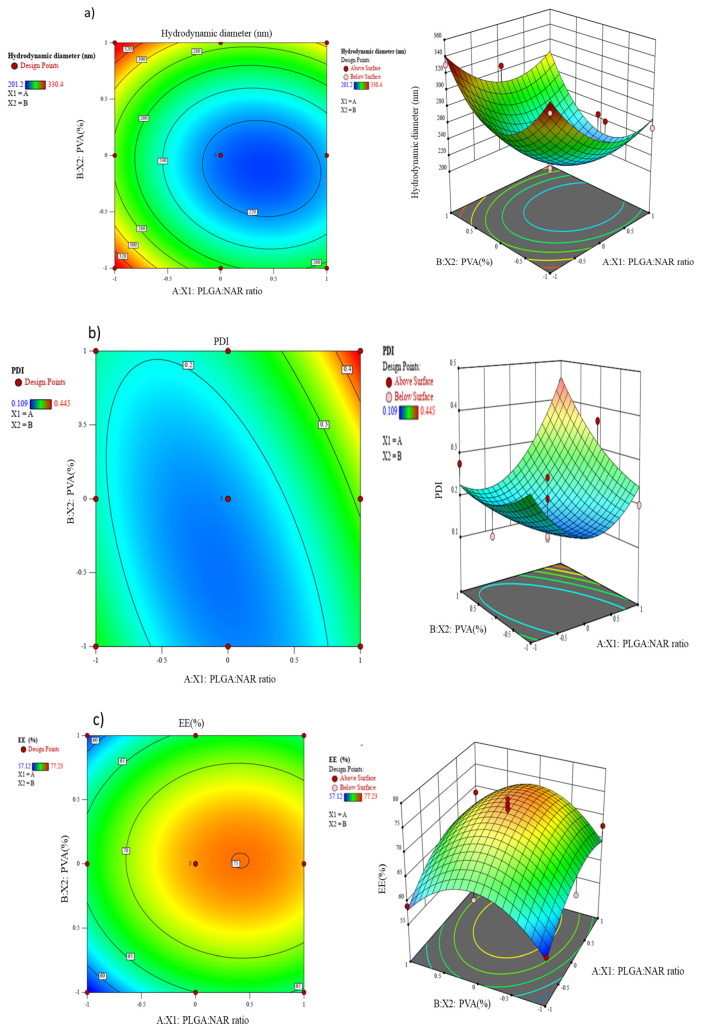
Contour and 3D surface response plots showing the effects of formulation variables on (**a**) hydrodynamic diameter, (**b**) polydispersity index (PDI), and (**c**) entrapment efficiency (EE) of PLGA nanoparticles.

The sign of the X_1_ and X_2_ coefficients indicates whether the factors had a positive or negative effect on the responses. The interactive effects of X_1_ and X_2_ variables on the responses were shown by the existence of interaction terms in polynomial equations and the importance of these factors. Furthermore, response surface and contour plots can be used to better illustrate these interacting effects (Figure 1). The elliptical shape of the contour lines confirmed a significant interaction between the PLGA: NAR ratio and PVA concentration. The lowest hydrodynamic diameter and PDI, and the highest entrapment, were achieved at the middle levels, suggesting that moderate levels of both variables are optimal.

Increase in PLGA: NAR ratio and PVA concentration from lower to middle levels demonstrated a significant decrease in hydrodynamic diameter and PDI and an increase in EE. At lower polymer concentrations, the emulsion droplets are not sufficiently stabilized. An increase in PVA and PLGA amounts to the optimum level contributed to stabilization of the emulsion, creating new interfaces and producing smaller, more uniformly sized nanoparticles. The smaller size of nanoparticles contributed to increased surface area, and the dense polymer matrix effectively trapped the drug, decreasing its diffusion into the external phase.

Further, an increase in PLGA: NAR ratio and PVA concentration from middle to higher levels demonstrated an increase in hydrodynamic diameter and PDI and a decrease in EE. Higher polymer concentration increased the viscosity of the organic phase, resulting in a dense polymer matrix, making it difficult to create smaller and evenly sized nanoparticles. The higher polymer concentration also contributed to steric hindrance due to long hydrophobic chains pushing the NAR out of the polymer matrix. Additionally, excess PVA could have resulted in micellization, causing the drug to leak out or partition into the external phase.

**Figure 2:**
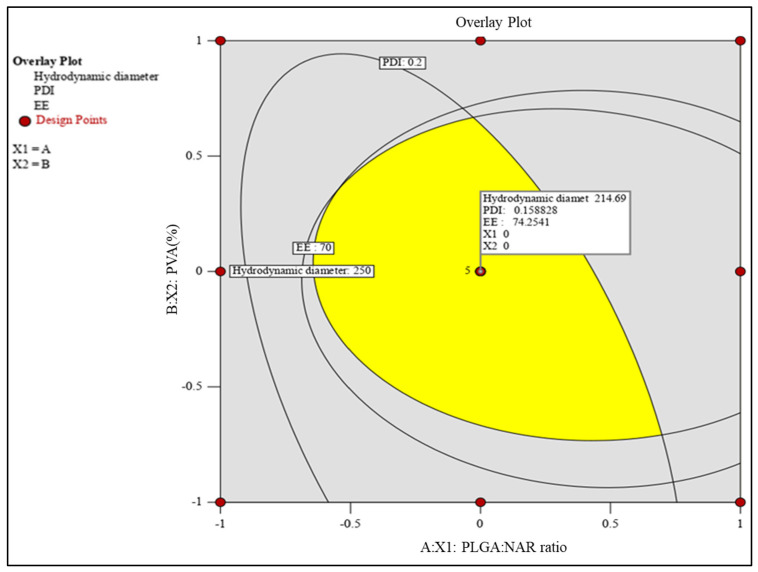
Overlay plot showing identified design space (yellow region) as a function of X_1_ (PLGA: NAR ratio) and X_2_ (PVA concentration).

The optimal or design space is seen in the overlay plot (Figure 2) as the yellow region. The variables, PLGA: NAR ratio of 3:1 and PVA concentration of 1%, were considered to be optimum for further study. The predictability of the model was established experimentally by comparing predicted and experimental values, which confirmed a prediction error below 10%. The optimum batches resulted in NP with a hydrodynamic diameter in the range of 201–210 nm, PDI from 0.1 to 0.2, and EE over 75%. The restricted size distribution, indicated by the PDI values (~0.1–0.2), points to consistent and stable nanoparticle production.

### 2.2. Characterization of PLGA NP and LC PLGA NP

#### 2.2.1. Size Analysis, Polydispersity Index, Morphology, and Zeta Potential

The hydrodynamic diameter of the optimized PLGA NP determined by dynamic light scattering was found to be 201.2 ± 1.3 nm with PDI 0.20 ± 0.03 and EE of 76.11 ± 2.1%. A lecithin-coated PLGA NP system for NAR LC PLGA NP was formulated using the optimized variable ranges and resulted in a hydrodynamic diameter of 308 ± 3.2 nm, PDI of 0.21 ± 0.05, and EE of 82.45 ± 4.8%. The particle size of NP is critical for colloidal stability, lung cell uptake, and pulmonary retention. The literature reports that smaller nanoparticles pass more easily through the three-dimensional mesh-like network of mucus and are taken up by epithelial cells via endocytosis, whereas particles above 500 nm are cleared by mucociliary clearance [36]. The size of LC PLGA NP was found to be higher than that of PLGA NP. Lecithin, being an amphiphilic phospholipid molecule, formed a hydrated layer around nanoparticles, increasing their hydrodynamic size, stabilizing the NP, and reducing drug leakage into the external phase, as demonstrated by higher EE.

**Figure 3:**
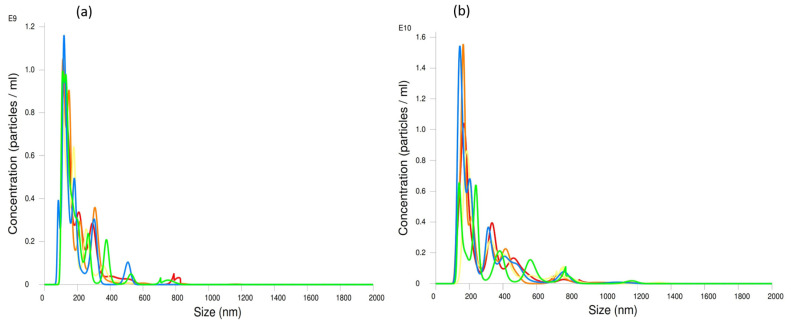
Size distribution profiles of (**a**) Naringin-loaded PLGA nanoparticles (PLGA NP) and (**b**) Naringin-loaded lecithin-coated PLGA nanoparticles (LC PLGA NP).

Nanoparticle tracking analysis further provided a detailed size distribution and concentration of NPs (Figure 3). PLGA NP exhibited a mode hydrodynamic diameter of 195.8 ± 4.7 nm, whereas LC PLGA NP showed a significantly larger mode hydrodynamic diameter of 302.8 ± 14.8 nm (Table 3). The mean hydrodynamic diameter of PLGA NP was 196.4 ± 3.8 nm, while that of LC PLGA NP was 298.2 ± 5.8 nm. The increase in hydrodynamic diameter following lecithin coating confirms the successful formation of a lipid layer around the PLGA core. Although PLGA NP falls closer to the lower end of the nanoscale range, the LC PLGA NP remained within the acceptable size range for nanoparticulate drug delivery systems.

Further insights into size distribution were obtained from percentile values, D10, D50, and D90. The D10 values for PLGA NP and LC PLGA NP were 109.4 ± 1.2 nm and 147.8 ± 6.2 nm, respectively, indicating that 10% of the particle population was below these sizes. The median size (D50) was 157.8 ± 4.2 nm for PLGA NP and 277.3 ± 15.2 nm for LC PLGA NP, highlighting a substantial shift toward larger size after lecithin coating. Similarly, the D90 values were 327.1 ± 12.4 nm for PLGA NP and 537.8 ± 37.1 nm for LC PLGA NP, demonstrating a broader size distribution and a higher proportion of larger particles in the lecithin-coated system. Overall, both formulations exhibited a slightly right-skewed size distribution, with an extended tail toward larger sizes. This skewness was more evident in LC PLGA NP, potentially reflecting the formation of larger lipid-coated nanoparticles or partial aggregation.

The comparative size distribution analysis (Table 3) confirms that lecithin coating increases the size of nanoparticles and broadens the size distribution while maintaining the nanoscale characteristics required for efficient lung-targeting applications. Additionally, the concentration per mL of PLGA NP was calculated to be 1.21 × 10^10^ ± 2.4 particles/mL, whereas for LC PLGA NP it was 8.93 × 10^9^ ± 4.4 particles/mL.

**Figure 4:**
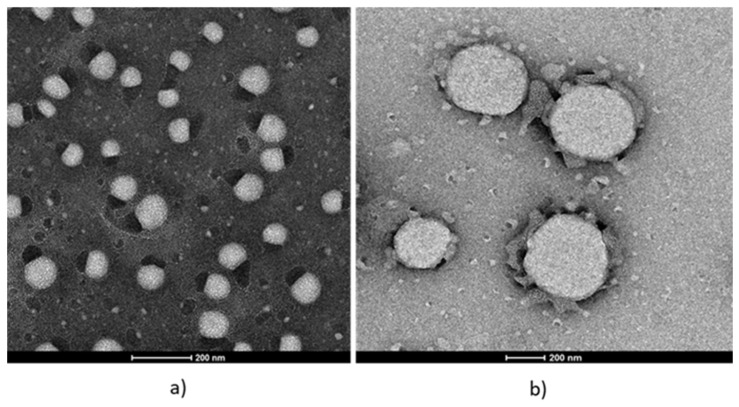
Transmission electron microscopy showing morphology of (**a**) Naringin-loaded PLGA nanoparticles (PLGA NP) and (**b**) Naringin-loaded lecithin-coated PLGA nanoparticles (LC PLGA NP).

The size, morphology, and internal structure of NP were further confirmed by TEM images (Figure 4). The PLGA NP images showed spherical-shaped nanoparticles with a size less than 100 nm, which is significantly lower than the size measurements made with DLS. The dynamic light scattering technique (by nanoparticle size analyzer or NTA) measures the hydrodynamic diameter of nanoparticles, which includes their surrounding hydration layer in size measurement, whereas TEM measures the size of nanoparticles in a dehydrated state, revealing the actual nanoparticle core. In contrast, TEM of LC PLGA NP demonstrated a larger size (more than 400 nm) compared to DLS. This behavior is especially observed in some lipid samples, including lecithin, where, during sample analysis, the lecithin coating flattens or collapses under vacuum, creating a higher apparent 2D size of NP compared to its 3D spherical diameter in solution [36,37].

The TEM micrographs demonstrated that both formulations exhibited a predominantly spherical morphology with a uniform size distribution. In the case of PLGA NP, the nanoparticles appeared as solid, dense structures with relatively smooth surfaces, indicative of the formation of a compact polymeric matrix. In contrast, LC PLGA NP displayed a distinct core–shell architecture, where a well-defined outer lecithin layer surrounded the hydrophobic PLGA core. This lipid shell was observed to be uniformly and intimately associated with the core, suggesting successful surface modification and effective coating.

#### 2.2.2. Zeta Potential

Zeta potential is another important parameter that determines the colloidal stability of NP. A zeta potential of −19.2 ± 3.4 mV for PLGA NP indicates moderate colloidal stability, where stabilization is mainly governed by steric effects rather than strong electrostatic repulsion. In contrast, a zeta potential of −28.1 ± 2.9 mV for LC PLGA NP reflects enhanced surface charge and improved electrostatic stability. The increased negative charge can be attributed to the presence of anionic phospholipids in lecithin, which expose negatively charged phosphate groups on the nanoparticle surface.

#### 2.2.3. Fourier-Transform Infrared Red Spectroscopy

**Figure 5:**
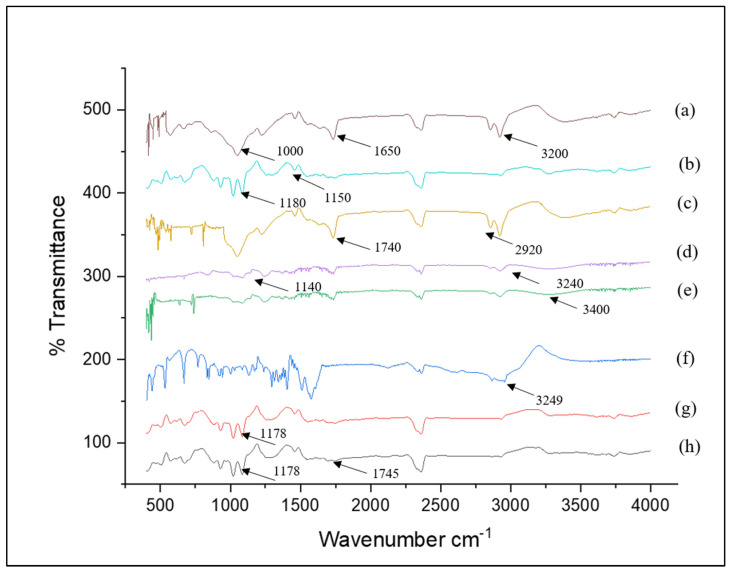
FTIR spectra of (**a**) Naringin (NAR), (**b**) PLGA, (**c**) SL, (**d**) Polyvinyl alcohol, (**e**) D-mannitol, (**f**) Physical mixture of all components, (**g**) NAR-loaded PLGA nanoparticles (PLGA NP), and (**h**) NAR-loaded lecithin-coated PLGA nanoparticles (LC PLGA NP).

The FTIR (Figure 5) analysis of NAR, PVA, PLGA, SL, their physical mixture (PM), and nanoparticles, PLGA NP and LC PLGA NP, was carried out to understand chemical interactions between different components of nanoparticle formulation. The FTIR spectrum of naringin exhibited (Figure 5a) a broad absorption band around 3200 to 3500 cm^−1^, corresponding to the stretching of phenolic –OH groups. Peaks observed near 3000 cm^−1^ were attributed to aromatic C–H. A prominent peak appeared around 1650 cm^−1^, corresponding to the carbonyl (C=O) group in the flavonoid structure. Additionally, strong bands observed around 1000 cm^−1^ were assigned to C–O and C–O–C stretching, confirming the presence of glycosidic bonds in naringin.

The FTIR spectrum of PLGA exhibited (Figure 5b) an absorption peak at around 1750–1760 cm^−1^, corresponding to the ester carbonyl (C=O) stretching vibration, which confirms the presence of ester linkages in the polymer backbone. Additionally, bands appearing between 1180 cm^−1^ corresponded to C–O–C stretching vibrations of the ester groups. Peaks at around 1450 cm^−1^ were assigned to C–H bending vibrations, confirming the structural integrity of the polymer. The FTIR spectrum of SL exhibited (Figure 5c) characteristic peaks corresponding to its phospholipid structure. A strong absorption band observed around 2920 cm^−1^ was attributed to the asymmetric and symmetric stretching vibrations of aliphatic –CH_2_ groups in the fatty acid chains. The prominent peak at approximately 1735 cm^−1^ corresponded to the ester carbonyl (C=O) stretching vibration, confirming the presence of phosphatidylcholine in the lecithin structure. The FTIR spectrum of PVA exhibited (Figure 5d) a broad absorption band at 3240 cm^−1^, corresponding to the stretching vibration of hydroxyl (–OH) groups involved in intermolecular hydrogen bonding. Furthermore, the peak at around 1140 cm^−1^ was attributed to C–O stretching of alcohol groups, confirming the characteristic structure of PVA. The FTIR spectrum of D-mannitol exhibited (Figure 5e) a broad absorption band at 3200 to 3600 cm^−1^, attributed to O–H stretching vibrations arising from extensive hydrogen bonding among hydroxyl groups.

The comparative analysis of the FTIR spectra of the physical mixture and the individual components, namely NAR, PLGA, PVA, and SL, demonstrated that the characteristic peaks of each constituent remained unchanged in the PM. This indicates the absence of any significant chemical interaction among the components.

In the PLGA NP(Figure 5g), all characteristic peaks were retained except those corresponding to SL, confirming the absence of lecithin and suggesting no chemical interaction within the formulation. In contrast, the LC PLGA NP exhibited (Figure 5h) a distinct absorption peak in the range of 1000–1180 cm^−1^, attributed to the C–O–C stretching vibrations of ester functional groups. Furthermore, no additional or shifted peaks were observed in the LC-PLGA NP formulation, indicating the absence of new chemical bond formation and confirming the physicochemical compatibility of the components [38].

#### 2.2.4. Differential Scanning Calorimetry

**Figure 6:**
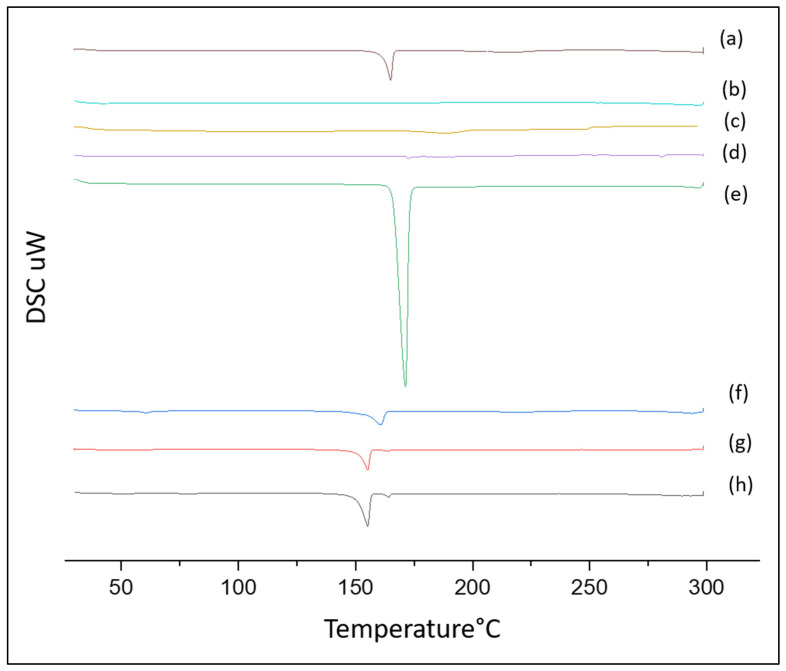
DSC graphs of (**a**) Naringin (NAR), (**b**) PLGA, (**c**) PVA, (**d**) SL, (**e**) D-mannitol, (**f**) Physical mixture of all components, (**g**) NAR-loaded PLGA nanoparticles (PLGA NP), and (**h**) NAR-loaded lecithin-coated PLGA nanoparticles (LC PLGA NP).

The DSC (Figure 6) thermograms were analyzed to evaluate the physical state of NAR during nanoparticle formulation. NAR showed a distinct endothermic peak at 160.5 °C, indicating its crystalline form and melting point. The DSC thermogram of PLGA showed a baseline shift in the range of 41 to 55 °C, indicating its glass transition temperature (Tg). PVA demonstrated a prominent endothermic peak at 188.5 °C, and D-mannitol indicated a sharp endotherm at 168.5 °C, confirming their crystalline nature. SL showed a melting endotherm at 172.3 °C, followed by several endothermic peaks indicating its decomposition. The physical mixture showed a sharp endothermic peak for D-mannitol due to its high proportion in the mixture. This endothermic peak of mannitol was broadened, and its intensity decreased in nanoparticle formulations due to lyophilization. This was further confirmed by XRD analysis. No sharp endothermic peak of NAR was identified in the NP formulation, indicating molecular entrapment of NAR during NP preparation. The sharp peak observed in the NP formulation may be attributed to the presence of D-mannitol.

#### 2.2.5. XRD

**Figure 7:**
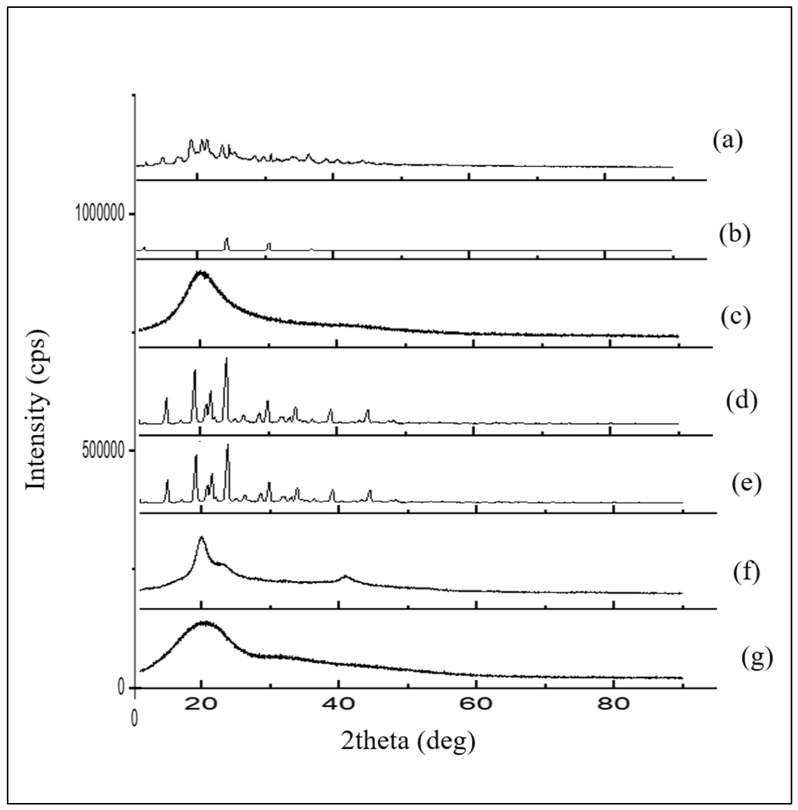
XRD patterns of (**a**) Naringin (NAR), (**b**) PLGA, (**c**) SL, (**d**) Mannitol, (**e**) Physical mixture of all components, (**f**) NAR-loaded PLGA nanoparticles (PLGA NP), and (**g**) NAR-loaded lecithin-coated PLGA nanoparticles (LC PLGA NP).

X-ray diffractograms (Figure 7) of NAR, PVA, and PLGA, SL, PM, PLGA NP, and LC PLGA NP were analyzed to study the changes in drug crystallinity during formulation. The NAR diffractogram showed high-intensity peaks at 5.97°–6.12°, 14.36°–14.7°, 24.01°–24.46°, characteristic of NAR. Mannitol indicated sharp characteristic peaks at 14.79°–15.18°, 18.66°–19.58°, 20.6°–21.71°, 23.4°–23.98°, confirming its crystalline nature. The characteristic peaks of mannitol and NAR were observed in the physical mixture. The X-ray diffractogram of the formulation indicated a decline in the intensity of the characteristic peaks of NAR, mannitol, and other ingredients present in the diffractogram of the physical mixture. This indicates the transformation of NAR and mannitol from a crystalline to an amorphous state during NP formulation and lyophilization.

#### 2.2.6. In Vitro Drug Release

**Figure 8:**
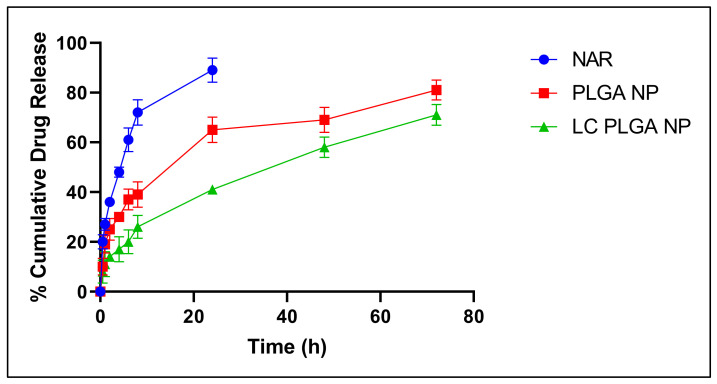
In vitro drug release profile of free Naringin (NAR), NAR-loaded PLGA nanoparticles (PLGA NP), and NAR-loaded lecithin-coated PLGA nanoparticles (LC PLGA NP) in phosphate buffer (pH 7.4).

The drug release (Figure 8) of NAR and PLGA NP was compared with that of the LC PLGA NP formulation, and the results are presented. NAR exhibited a rapid and uncontrolled release pattern, characterized by a pronounced initial burst. Despite its poor solubility, approximately 60.1 ± 4.3% of the drug was released within the first 6–8 h, followed by a cumulative release of 90.5 ± 3.9% by 24 h. Thereafter, no significant increase in cumulative release was observed up to 72 h. PLGA NP showed a clear biphasic release profile, with a burst release at the beginning and a steady release phase afterwards. An initial release of approximately 40.7 ± 5.1% was observed within the first 12 h, which may be attributed to the presence of drug molecules adsorbed or weakly bound to the nanoparticle surface. This was followed by a gradual and controlled release phase governed primarily by drug diffusion through the PLGA matrix and progressive polymer erosion. The cumulative drug release from PLGA NP reached approximately 81.68 ± 4.9% at 72 h, indicating a sustained release compared to pure naringin.

LC PLGA NP exhibited the most prolonged and controlled drug release profile compared to NAR and PLGA NP. The initial burst release was further reduced to approximately 15.8 ± 4.1%, confirming the role of the lecithin coating as an additional diffusion barrier. Drug release from LC PLGA NP proceeded in a slow and sustained manner throughout the study period, with cumulative release reaching approximately 71.4 ± 3.2% at 72 h. The delayed release behavior is attributed to the combined effects of restricted drug release through the lecithin shell and the gradual degradation of the PLGA core. The sustained release of NP is desirable in targeted drug delivery, as it minimizes premature drug release before reaching target cells. Based on their size and surface characteristics, NPs are taken up by target cells via endocytosis, and the drug is released within the cells after digestion of the NP by lysosomal enzymes. The model fitting for dissolution data indicated drug release was governed by diffusion from the polymer matrix, which was further confirmed by an n value of 0.5 for the Korsmeyer–Peppas model.

#### 2.2.7. Cell Culture Studies

##### Cell Viability Study

**Figure 9:**
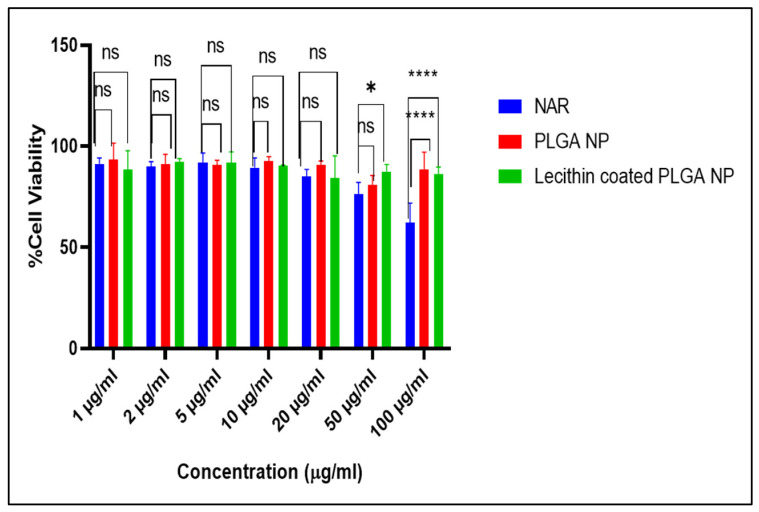
In vitro cell viability of A549 cells after treatment with free NAR, NAR-loaded PLGA NP, and NAR-loaded LC PLGA NP at various concentrations. Values represent an average ± standard deviation (*n* = 3). Significant difference between treatments is denoted by * *p* value< 0.05, **** *p* value < 0.0001, & ns-non significant.

The cytotoxic potential (Figure 9) of free NAR, PLGA NP, and LC PLGA NP was evaluated using the MTT assay in the human lung epithelial A549 cell line. The MTT assay measures cellular metabolic activity based on the ability of mitochondrial succinate dehydrogenase in viable cells to reduce yellow tetrazolium salt (MTT) to insoluble purple formazan crystals. The amount of formazan formed is directly proportional to the number of metabolically active cells, serving as an indicator of cell viability. A549 cells were treated with increasing concentrations of NAR, PLGA NP, and LC PLGA NP (1–100 μg/mL). For NAR, cell viability remained close to 90% at concentrations up to 20 μg/mL, while at 50 μg/mL and 100 μg/mL, viability decreased to 76% and 62%, respectively. These observations are consistent with literature reports, where the IC50 for naringenin (the aglycone of NAR) ranges from 35–100 μg/mL, and for NAR, from 80–220 μg/mL [39,40].

Importantly, all tested nanoparticle formulations exhibited minimal cytotoxicity across the concentration range, with cell viability remaining near 90% even at 100 μg/mL. These results indicate that both free NAR and nanoparticle formulations are non-toxic to lung epithelial cells within the studied concentrations. The absence of cytotoxic effects demonstrates the excellent biocompatibility of PLGA NP and lecithin-coated systems, in agreement with previously reported findings for PLGA and lipid-based nanocarriers [41].

##### Cellular Uptake Studies

**Figure 10:**
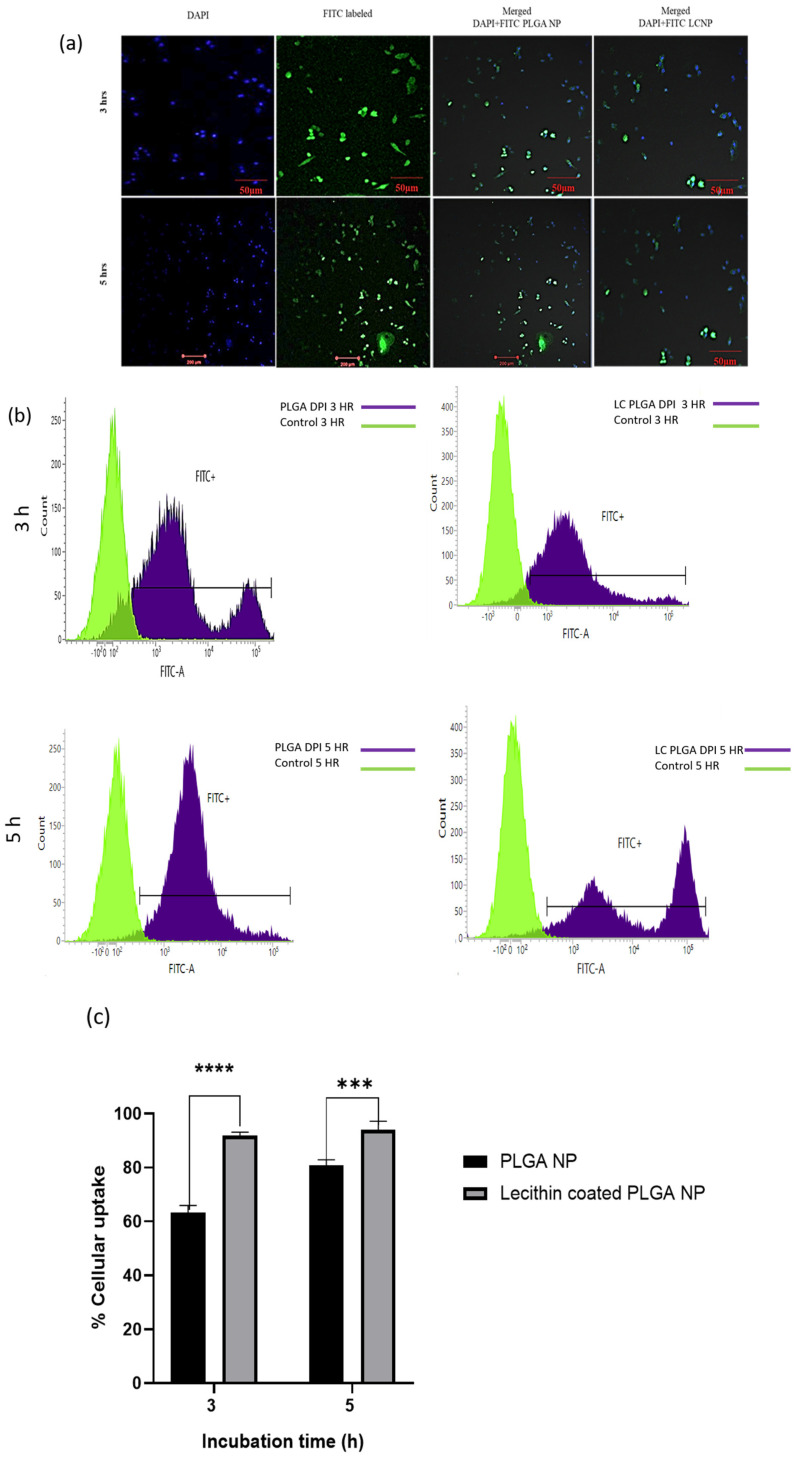
In vitro cell uptake analysis in A549 cells. (**a**) Confocal laser scanning microscopy (CLSM) images showing the internalization of FITC-labelled PLGA NPs and LC PLGA NPs after 3 h and 5 h of incubation (Blue: DAPI-stained nuclei; Green: nanoparticles (**b**) Flow cytometry histograms illustrating the shift in fluorescent intensity indicating nanoparticle uptake over time. (**c**) Quantitative percentage cellular uptake from flow cytometry data. Values represent an average ± standard deviation (*n* = 3). Statistical significance between treatments denoted as *** *p* value < 0.001 and **** *p* value < 0.0001.

The potential of a nanoparticle system to target a drug was determined by two factors: the intracellular release of the drug and the uptake of NP internalization into the cell. PLGA NP and LC PLGA NP uptake by the A549 lung epithelial cell line was measured using cell uptake studies. Confocal microscopy and flow cytometry were used to quantify and qualitatively assess the cells’ uptake of both nanoparticle types. The results are presented in (Figure 10). Confocal laser scanning microscopy images confirmed the incorporation of fluorescein isothiocyanate (FITC), a fluorescent derivative of fluorescein, within the nanoparticulate system. FITC was successfully encapsulated within the nanoparticles, as evidenced by its distinctive fluorescence with an excitation wavelength of 488 nm and an emission wavelength of 517 nm. Nuclei are stained blue by DAPI, a blue fluorescein derivative with an emission wavelength of 461 nm and an excitation wavelength of 358 nm. After five hours of incubation, FITC-loaded LC PLGA NPs showed a higher percentage of internalization than FITC-loaded PLGA NPs. Flow cytometry results further confirmed these observations. After three and five hours of incubation, the percentage of fluorescence retained in the cells was 83.8 ± 2.3% and 92.6 ± 4.5% for polymeric NP and 92.9 ± 2.5% and 96.4 ± 4.8%, respectively, for LC PLGA NP. This demonstrates that compared to polymer nanoparticles alone, LC PLGA NP significantly improves cell internalization (**** *p* value < 0.0001 & *** *p* <0.001). The lecithin coating imparts surface hydrophilicity and mimics the shape of the cell membrane, which would have made it easier for these NP to be uptaken. As demonstrated in previous research, coating nanoparticles with lecithin or other comparable biomimetic lipids increases cell uptake [42]. Furthermore, the application of a hydrophilic surface coating on nanoparticles enhances their cellular retention and the intracellular accumulation of encapsulated bioactive agents. This effect is primarily attributed to the reduced interaction of nanoparticles with efflux transporters, such as P-glycoprotein, thereby minimizing drug expulsion and improving cellular uptake efficiency [43,44].

##### In Vitro Oxidative Stress Assay

**Figure 11:**
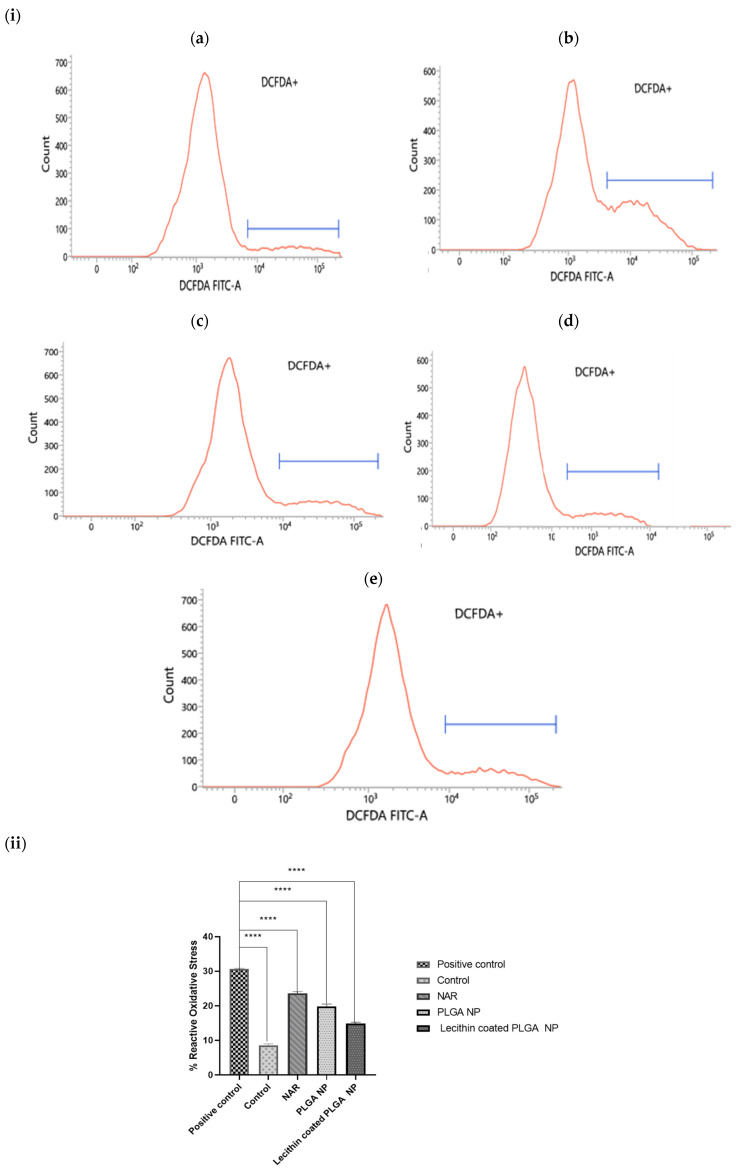
Intracellular Reactive Oxygen Species (ROS) levels in A549 cells. (**i**) Representative flow cytometry histograms showing the fluorescence intensity of ROS indicator after treatment with: (**a**) Positive control, (**b**) Control (untreated), (**c**) Free Naringin (NAR), (**d**) NAR-loaded PLGA NPs, and (**e**) NAR-loaded LC PLGA NPs and (**ii**) Quantitative analysis of percentage ROS generation relative to the control group. Values represent an average ± standard deviation (*n* = 3). Asterisks denote statistical significance **** *p* value < 0.0001.

In the reactive oxygen stress (ROS) assay, A549 lung epithelial cells in the control group exhibited basal ROS levels, indicating normal redox homeostasis. Treatment with NAR, PLGA NP, and LC PLGA NP resulted in a significant reduction in intracellular ROS levels compared with the positive control (Figure 11) (**** *p* value < 0.0001). Among the treated groups, LC PLGA NP demonstrated a markedly significant reduction in ROS levels compared to NAR and PLGA NP (Figure 11) (*** *p* value < 0.001 and * *p* value <0.05). The pronounced decrease in ROS observed with LC PLGA NP treatment indicated efficient intracellular antioxidant activity and effective interaction with lung epithelial cells. Overall, these findings demonstrate that LC PLGA NP effectively reduced oxidative stress in A549 lung epithelial cells under inflammatory conditions, supporting their potential for lung-targeted antioxidant therapy [45].

##### In Vitro Anti-Inflammatory Cytokines Study

**Figure 12:**
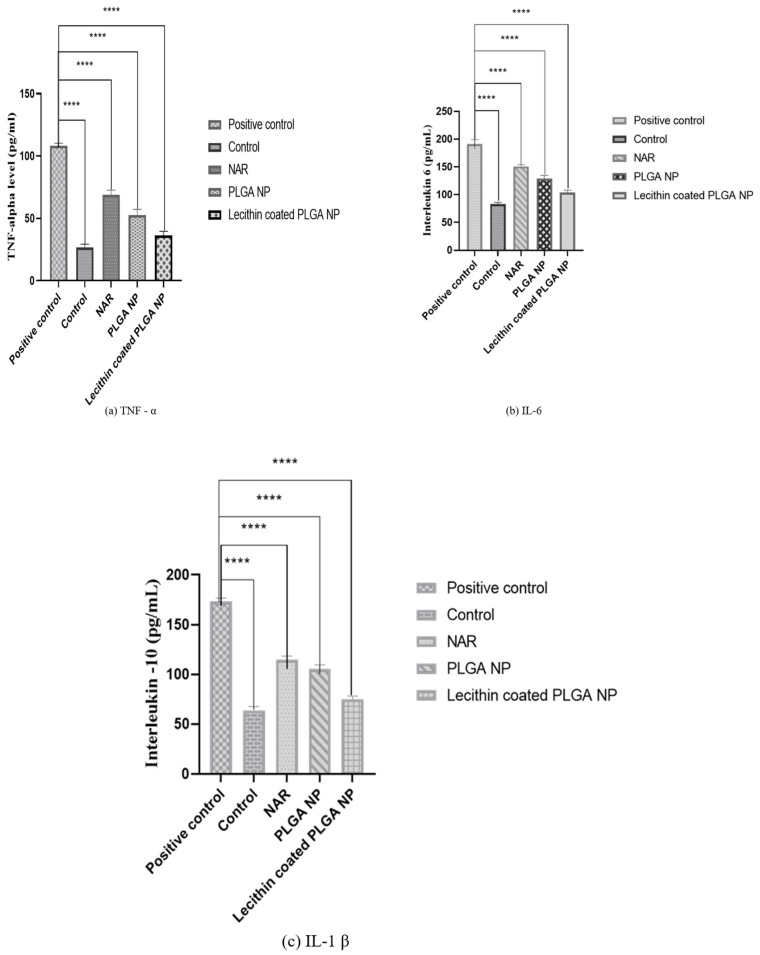
In vitro anti-inflammatory activity of Free Naringin (NAR), NAR-loaded PLGA NPs, and NAR-loaded LC PLGA NPs assessed by levels of pro-inflammatory cytokines, (**a**) TNF-α, (**b**) IL-6, and (**c**) IL-1β measured in LPS-treated A549 cells. Data represent mean ± SD (*n* = 3). Significant differences between groups are indicated by asterisks **** *p* value < 0.0001.

LPS stimulation significantly increased the levels of pro-inflammatory cytokines, IL-6, IL-1β, and TNF-α in comparison with untreated control cells, confirming successful induction of an inflammatory response (Figure 12) (**** *p* value < 0.0001). Treatment with NAR, PLGA NP, and LC PLGA NP significantly reduced the levels of IL-1β, TNF-α, and IL-6 compared to the LPS-treated inflammatory group. Among the tested formulations, LC PLGA NP produced the most pronounced suppression of cytokine secretion. Treatment with NAR, PLGA NP, and LC PLGA NP effectively attenuated the LPS-induced inflammatory response. The superior anti-inflammatory activity observed with LC PLGA NP was attributed to improved cellular interaction and enhanced intracellular delivery of NAR. The presence of the lecithin coating likely enhanced mucus penetration and improved cell uptake by inflamed cells, resulting in more effective suppression of cytokine secretion. In contrast, free NAR and uncoated PLGA NP exhibited comparatively lower efficacy, possibly due to limited cellular uptake and reduced intracellular drug availability [46].

#### 2.2.8. Angiogenesis Assay [Yolk Sac Membrane (YSM)]

**Figure 13:**
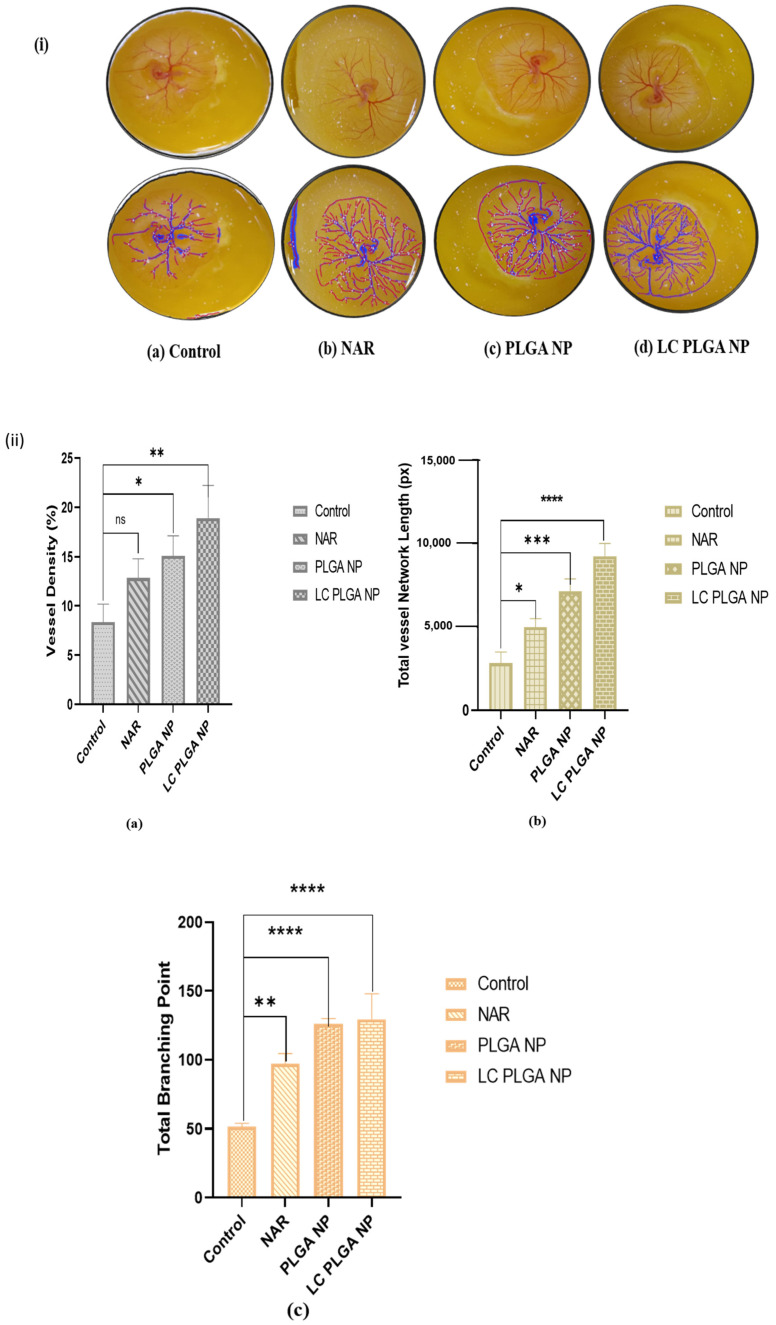
Angiogenic potential of Naringin formulations. (**i**) Representative microscopic images of the vascular network in YSM assay after treatment with (**a**) Control, (**b**) Free Naringin, (**c**) NAR-loaded PLGA nanoparticles, and (**d**) NAR-loaded lecithin-coated PLGA nanoparticles. (**ii**) Quantitative morphometric analysis of the vascular architecture (**a**) Vessel density, (**b**) Total vessel network length, and (**c**) Number of total branching points. Data expressed as mean ± SD (*n* = 3). Statistical significance is denoted by **** *p* value < 0.0001, *** *p* value < 0.001, ** *p* value < 0.01. * *p* value < 0.05 and ns- non-significant.

The angiogenic potential and tissue remodeling ability of the developed NP formulation were evaluated using the egg yolk sac membrane (YCM) model (Figure 13). Following application of the formulation, a well-organized and dense vascular network was observed compared with the control. Quantitative analysis (Figure 13) showed a statistically significant increase in vessel density, total vessel network length, and the number of branching points, with no hemorrhage, vessel rupture, or inflammatory damage, confirming vascular safety. The angiogenic response indicated favourable endothelial compatibility, suggesting potential retention within pulmonary capillaries and enhanced lung targeting. Overall, the egg yolk sac angiogenesis assay provided supportive evidence of the formulation’s lung-relevant vascular compatibility and endothelial-targeting potential.

## 3. Materials and Methods

### 3.1. Materials

NAR and Fluorescein isothiocyanate (FITC) were procured from Sigma-Aldrich (Mumbai, Maharashtra, India). PLGA (50:50) Parenteral Grade/Medical Grade, Molecular Weight (M.W.) 7000–17,000 Da was procured from Nomisma Health Care Pvt. Ltd., Vadodara, Gujarat, India. PVA Cold (M.W. 1,25,000), D-mannitol and SL were procured from Himedia, Mumbai, Maharashtra, India. DMSO, formaldehyde, and acetone were obtained from Research Lab Chem Industries, Mumbai, Maharashtra, India. For the YSM assay, eggs were procured from Venkateshwara Hatcheries Private Limited, Pune, Maharashtra, India. The human lung epithelial cell line A549 (NCCS-AT-011), derived from alveolar adenocarcinoma, was used as an in vitro model to evaluate pulmonary cytotoxicity and anti-inflammatory activity and was procured from NCCS (National Centre for Cell Science), Pune, Maharashtra, India. The cell culture medium Dulbecco’s Modified Eagle Medium (DMEM), Trypsin EDTA used for cell detachment, Fetal bovine serum (FBS) used for cell nutrition and growth, phosphate buffer saline (PBS) used for washing the cells, and 3-(4,5-dimethylthiazol-2-yl)-2,5-diphenyltetrazolium bromide (MTT), and 4,6-diamino-2-phenylindole (DAPI) were purchased from Himedia, Mumbai, Maharashtra, India. tert-butyl hydroperoxide (tBHP) was procured from Thermo Fisher Scientific, Waltham, Massachusetts, United States, and 2′,7′-dichlorodihydrofluorescein diacetate (DCFDA) from Sigma-Aldrich, Bangalore, Karnataka, India for cell line experiments. In cell culture studies, a flow cytometer (BD FACS Lyric, Becton, Dickins, United States of America), a confocal microscope (Zeiss LSM-710, Oberkochen, Baden-Württemberg, Germany), a microplate reader (CYT5M, BioTek Cytation 5, Winooski, Vermont, United States of America) were used. TNF-α (Cat. No. KB3145), IL-6 (Cat. No. KB3068), and IL-1β (Cat. No. KLR0119) ELISA kits were procured from KRISHGEN Biosystems, Mumbai, Maharashtra, India.

### 3.2. Methods

#### 3.2.1. Preparation of NAR-Loaded PLGA NP by the Emulsion-Solvent Evaporation Technique

PLGA NP loaded with NAR were prepared by the emulsion solvent evaporation method. The organic phase was formed by dissolving PLGA and NAR in 10 mL of acetone at ratios of 2:1, 3:1, and 4:1. This organic phase was then added dropwise to 40 mL of an aqueous phase containing PVA at concentrations of 0.5%, 1%, and 1.5% (*w*/*v*) under high-speed homogenization for 30 min to form an emulsion. The emulsion was further subjected to probe sonication for 15 min [47]. The optimized NP formulation was coated with 0.1% (*w*/*v*) SL. Briefly, lecithin was dissolved in the aqueous phase and incorporated using the same preparation procedure as employed for the uncoated formulation, resulting in the formation of LC PLGA NP [48]. For characterization, the prepared nanoparticle was subjected to lyophilization (freeze-drying) to obtain a dry, free-flowing powder. Briefly, mannitol (5% *w*/*v*) was added to the NP, which were further frozen at −80 °C for 24 h to ensure complete solidification. The frozen samples were then transferred to a Lyophilizer (Martin Christ, Alpha-2-4 LCS, Osterode am Harz, Lower Saxony, Germany) and subjected to Primary drying for 20 h, and secondary drying was done for 4 h at 0.104 mbar pressure. Upon completion of the lyophilization cycle, the dried nanoparticle cake was collected and stored in airtight containers at 4 °C until further use.

#### 3.2.2. Optimization of NAR-Loaded PLGA Nanoparticles by Face Centred Central Composite Design (CCD)

For the optimization of a PLGA NP formulation, a central composite design using Design-Expert Software version 13 (Minneapolis, MN, USA) was applied. In a CCD design, two components were used as independent variables: PLGA: NAR ratio (X_1_) and PVA concentration (X_2_) (Table 1). Preliminary experiments were performed to find the levels for each variable, evaluating their ability to form a nanoparticle. The optimization focused on the following responses: Hydrodynamic diameter (Y1), Poly dispersity index (PDI) (Y2), and % Entrapment efficiency (Y3). Design-Expert Software version 13 (Stat-Ease Inc., Minneapolis, MN, USA) was used to develop and assess the experimental runs. Mathematical models were created from the experimental data using analysis of variance (ANOVA) [49]. The optimized nanoparticle formulation was coated with 0.1% (*w*/*v*) SL using the same preparation procedure as that of PLGA NP to obtain LC PLGA NP.

#### 3.2.3. Characterization of Nanoparticles

##### Entrapment Efficiency (EE)

The NAR entrapped in the PLGA NP and LC PLGA NP was determined by separating and analyzing the free NAR from the entrapped one using the centrifugation method. Briefly, the nanoparticles were placed in a Pierce Protein Concentrator tube (Thermo Scientific, Waltham, MA, USA) and centrifuged at a speed of 15,000 rpm for 15 min (Remi, RM-03 Plus centrifuge, Mumbai, Maharashtra, India). The amount of unentrapped drug present in the filtrate was quantified using a Shimadzu LC-10AT, Kyoto, Kyoto Prefecture, Japan, with a sample injector fitted with a 20 µL loop and a photodiode array (PDA) detector. The C18 column (Kromasil 100-5-C18 with dimensions of 4.6 × 250 mm with 5 µm) was the stationary phase used. The mobile phase was composed of a 30:70 (*v*/*v*) ratio of acetonitrile to potassium phosphate buffer (25.0 mM, pH 3 ± 0.1, containing 0.2% triethylamine, pH adjusted with diluted orthophosphoric acid). The flow rate of the mobile phase was 1.0 mL/min. At 284 nm, the eluent was identified while the column temperature was kept at 25 °C. Percent EE was calculated as follows. The experiment was carried out in triplicate, and the mean measurement was used [50].

$$EE\;\left(\%\right)=\frac{Total\;Drug-Unentraped\left(free\right)\;Drug}{Total\;Drug}\times 100$$

##### Size Distribution, Polydispersity Index, and Zeta Potential

The characterization of PLGA NP and LC PLGA NP was performed by evaluating the hydrodynamic diameter of NP, polydispersity index (PDI), and zeta potential. A nanosize analyzer based on dynamic light scattering (DLS) (Horiba, SZ-100Z, Kyoto, Kyoto Prefecture, Japan) was used to measure hydrodynamic size and PDI. At a fixed scattering angle of 90°, measurements were taken at 25°C. Before analysis, the nanoparticle dispersion was prepared by dispersing 10 mg of nanoparticle powder in 20 mL of 7.4 pH phosphate buffer. For hydrodynamic diameter analysis, the dispersion was further diluted (1 to 5) with Millipore water to reduce multiple scattering. A zeta cell was used to determine the zeta potential after ten dilutions of the NP sample with a conducting solution. Every measurement was performed in triplicate. The size distribution and concentration of the optimized nanoparticles were further confirmed by NTA analysis using NanoSight Software Version 3.4 Build 3.4.4 (Malvern, Wiltshire, United Kingdom). The diluted samples were gently vortexed to ensure homogeneity and were analyzed immediately to prevent aggregation or degradation. Samples were introduced into the chamber of a NanoSight system under sterile conditions. The laser and camera settings were optimized to capture the Brownian motion of the particles, and 60-s videos were recorded per sample. These recordings were processed using NTA Software Version 3.4 Build 3.4.4, which calculated the size distribution and concentration of particles based on the Stokes–Einstein equation. The dilution factor was considered to automatically adjust the concentration readings to reflect the original, undiluted sample.

##### Transmission Electron Microscope (TEM)

The surface morphology of the polymer nanoparticles was examined using TEM, JEM 2100 Plus (Tokyo, Tokyo Metropolis, Japan). The negative staining with 2% phosphotungstic acid (PTA). One drop of the PLGA NP and LC PLGA NP formulation was applied to copper grids coated with carbon, and then 2% PTA was added. The sample was allowed to interact with the staining agent for 3 min, and the excess stain was removed. Before being examined with a JEM 2100 Plus transmission electron microscope, the grid was air-dried. The lipid-coated polymeric nanoparticles were imaged at an accelerating voltage of 200 kV for improved visibility and structural characterisation. After locating and focusing the region of interest at low magnification, the inspection was gradually increased to (100,000 X) to collect thorough morphological information [51].

##### Fourier-Transform Infrared Red Spectroscopy (FTIR)

FTIR spectra of NAR, PLGA, PVA, SL, D-mannitol, PM, PLGA NP, and LC PLGA NP were obtained using a Bruker ALPHA II spectrometer (Ettlingen, Baden-Württemberg, Germany) with a Platinum-ATR attachment. The spectra were studied from 500 to 4000 cm^−1^ [52].

##### Differential Scanning Calorimetry (DSC)

Differential scanning calorimetry (Hitachi, DSC 7020, Oyama, Tochigi, Japan) was used to analyse the melting and crystallization behaviors of NAR, PLGA, PVA, SL, D-mannitol, PM, PLGA NP, and LC PLGA NP. Precisely weighed samples (2–4 mg) were enclosed in aluminum pans and analyzed under a nitrogen atmosphere maintained at a purge flow rate of 20 mL/min. Thermal analysis was performed at a constant heating rate of 10 °C/min across a temperature range from 30 °C to 350 °C [53].

##### X-Ray Diffraction (XRD)

The X-ray diffraction analysis was performed to identify the drug’s physical state and changes occurring during nanoparticle formulation. A sample was placed on a horizontal quartz glass holder plate and exposed to Cu Kα radiation with a wavelength of 1.5405 Å to use powder X-ray diffraction (PXRD) (Rigaku, Miniflex 600, Akishima, Tokyo, Japan) to analyse the crystalline behavior of NAR, PLGA, PVA, SL, D-mannitol, PM, PLGA NP, and LC PLGA NP. Under the settings of voltage 40 kV and current 15 mA25, the diffraction data were gathered at a scan rate of 1°/min throughout a 2θ range of 20° to 90° [54].

##### In Vitro Drug Release

In vitro drug release studies of PLGA NP and LC PLGA NP were conducted using a USP dissolution test apparatus II. Nanoparticle equivalent 7.5 mg of NAR in 5 mL of buffer medium, resulting in a final concentration of 1.5 mg/mL. 5 mL of NP dispersion was filled in a dialysis bag (Molecular Weight 12,000 to 14,000 Daltons (12–14 kDa, Himedia, India) and cutoff Diameter (Pore Size): 2.4 nanometers (nm)) and immersed in 250 mL of phosphate buffer (pH 7.4). The dissolution study was performed at 37 ± 5 °C with a paddle speed of 50 rpm. Samples (5 mL) were collected at predetermined time intervals (0.5 h, 1 h, 2 h, 4 h, 6 h, 8 h, 24 h, 48 and 72 h), appropriately diluted, and analysed using a Shimadzu LC-10AT HPLC system. The withdrawn volume was replaced with a fresh 5 mL buffer to ensure perfect sink conditions. Analysis was carried out by the previously validated HPLC method using similar conditions as mentioned in “Section Entrapment Efficiency (EE)” [55,56]. The linearity was established in the range 0.1 µg/mL to 20 µg/mL. The readings were recorded in triplicate, and results were reported as mean ± SD.

#### 3.2.4. Cell Culture Studies

Human lung epithelial A549 cells were cultivated in Dulbecco’s Modified Eagle Medium (DMEM) supplemented with 10% fetal bovine serum (FBS). Cells were kept in a CO_2_ incubator (Thermo Scientific, USA) at 37 ± 0.5 °C in a humidified environment (95% relative humidity) with 5% CO_2_. To maintain ideal development conditions, the culture media were replaced every two to three days. The cells were trypsinized using a 0.25% trypsin–EDTA solution once the cells reached around 90% confluency. Cells were seeded onto 8-well chamber slides and 6-well plate for qualitative and quantitative cellular uptake analysis using confocal laser scanning microscopy and flow cytometry, respectively, and into 96-well plates for cell viability using the MTT assay [57].

##### Cell Viability Study

The viability of lung epithelial A549 cells was evaluated using the MTT assay. Cells were first seeded in 96-well plates at a density of 2 × 10^5^ cells per well in 100 μL of culture media, and they were then incubated for 24 h to allow cell adhesion. The cells were then treated with different concentrations of NAR, PLGA NP, and LC PLGA NP (1 μg/mL to 100 μg/mL). Before being diluted into the medium, the NAR was first dissolved in phosphate buffer. The stock solutions of PLGA NP and LC PLGA NP were prepared by dissolving the formulations in phosphate buffer, followed by appropriate dilution with the culture medium to obtain the desired concentrations. Subsequently, 20 μL of MTT solution (5 mg/mL) was added to each well, and the cells were incubated at 37 °C for 4 h. After incubation, the supernatant was carefully removed, and 100 μL of dimethyl sulfoxide (DMSO) was added to each well to solubilize the formed formazan crystals. This was followed by 15 min of incubation and shaken in the dark. Finally, the microplate reader (Cytation5, CYT5M BIO-TEK, USA) measured the absorbance from each well at 560 nm [58,59].

##### Cellular Uptake

A549 lung epithelial cells were seeded at a density of 5 × 10^5^ cells/well in a 6-well cell culture plate and an 8-well chamber slide. At a final concentration of 10 μg/mL, FITC-labeled PLGA NP and LC PLGA NP were given to the cells. At a temperature of 37 ± 0.5 °C, treatment was carried out for three and five hours. After (3 and 5 h) intervals, 4% paraformaldehyde solution was used to fix the cells. The cells in each well were incubated with 0.5 μL of 4′-6-diamidino-2-phenylindole (DAPI) (0.5 mg/mL) for 15 min, and the cells were then washed 3X using phosphate-buffered saline (PBS). Trypsinized cells were quantitatively analyzed using flow cytometry (FACS-Lyric cytofluorometer, USA) and qualitatively analyzed using a confocal microscope (Zeiss, LSM-710, Germany) [60].

##### In Vitro Oxidative Stress Assay

Lung epithelial A549 cells were seeded at a density of 5 × 10^3^ cells per well in 6-well cell culture plates and incubated for 24 h at 37 °C in a humid environment with 5% CO_2_ to allow cell adhesion. After incubation, treatment was given by oxidative stress inducer, i.e., (50 μM) tert-butyl hydroperoxide (tBHP). After a 24 h incubation period, the culture medium was carefully removed, and the cells were gently washed with PBS to remove residual medium. Subsequently, the cells were incubated with 10 µM 2′,7′-dichlorodihydrofluorescein diacetate (DCFDA) prepared in serum-free media at 37 °C for 30 min under dark conditions to facilitate intracellular staining [61]. After staining, the excess dye was removed, and the cells were washed 2X with PBS. The cells were subsequently harvested by gentle trypsinization, neutralized with complete medium, and centrifuged at 2000 rpm for 10 min. The resulting cell pellet was resuspended in 500 µL of PBS and kept protected from light until further analysis. Further quantitative analysis was done by flow cytometry [62].

##### In Vitro Anti-Inflammatory Cytokines Study

Lung epithelial A549 cells were seeded in 6-well cell culture plates at a density of 5 × 10^5^ cells per well and allowed to attach for 24 h at 37 °C in a humid atmosphere containing 5% CO_2_. Following attachment, the cells were treated with an inflammatory stimulus, 1 µg/mL lipopolysaccharide (LPS) (15 µL) in each well, and formulations were administered simultaneously. The cells were then incubated for 24 h under standard culture conditions. After the treatment period, the culture medium was carefully aspirated to remove residual serum and secreted cytokines, and the cells were gently rinsed once with sterile PBS [63]. Fresh serum-free culture medium was subsequently added, and the cells were further incubated for an appropriate conditioning period to allow the secretion of cytokines into the medium. After incubation, the conditioned media were carefully collected without disturbing the cell monolayer and transferred into sterile microcentrifuge tubes. The samples were centrifuged at 1000–2000 rpm for 10 min in a cooling centrifuge (at 4 °C) to remove any cellular debris. The clarified supernatant was then aliquoted and preserved at −80 °C until further analysis [64]. The analysis was done by ELISA assay for TNF-α, IL-6, and IL-1β. The concentrations of TNF-α, IL-1β, and IL-6 were quantified using commercially available sandwich ELISA kits. Briefly, for TNF-α, standards and samples were added to the wells and incubated at 37 °C, followed by sequential incubation with biotinylated TNF-α antibody and streptavidin–HRP conjugate, with intermediate washing steps. For IL-1β and IL-6, plates were incubated with the respective biotinylated antibodies, followed by streptavidin–HRP conjugate. In all assays, TMB substrate was added for colour development, and the reactions were terminated using the terminator. Absorbance was recorded at 450 nm using a microplate reader.

#### 3.2.5. In Vitro Angiogenesis Activity by Yolk Sac Membrane (YSM) Assay

In a humidified incubator, fertilized chick eggs were kept at 37 °C for 48 h. After incubation, a small hole was created on the egg’s blunt end, and 3–4 mL of albumin was removed. Furthermore, treatment was given of 10 µg/mL of NAR, PLGA NP, and LC PLGA NP. After sealing eggs with transparent adhesive tape, the eggs were incubated for a further 48 h at 37 °C in humid conditions. After incubation for 48 h, pictures of blood vessel sprouting were taken and quantitatively examined using the Wim CAM online analysis tool (Wim CAM, Wimasis, Version 1.1, Munich, Germany). Total vessel network length, vessel density, and total number of vessel segments were among the criteria assessed [65,66,67].

## 4. Statistical Data Analysis

The data from in vitro cell culture studies and in vitro angiogenesis activity were analyzed using one-way analysis of variance (ANOVA) followed by Tukey’s multiple comparison test. Results were expressed as mean ± standard deviation (SD). Statistical analysis was performed using GraphPad Prism (Version 8.0.2). Statistical significance was defined as * *p* < 0.05, ** *p* < 0.01, *** *p* < 0.001, and **** *p* < 0.0001.

## 5. Conclusions

In this study, PLGA NP and LC PLGA NP were developed using the solvent evaporation method. The optimized nanoparticles demonstrated a size below 300 nm, high entrapment efficiency, and sustained drug release. LC PLGA NP demonstrated enhanced cellular uptake, reduced oxidative stress, and a reduction in pro-inflammatory cytokines (TNF-α, IL-1β, IL-6) compared to PLGA NP. Furthermore, these nanoparticles can be formulated as dry powders using a suitable diluent for deep lung targeting and therapeutic application. Overall, the developed lecithin-coated PLGA nanoparticles of NAR provide an effective strategy to enhance cellular uptake and therapeutic efficacy in inflammatory lung conditions.

## Figures and Tables

**Table 1:** Experimental Design Based on Face-Centred Central Composite Design and Corresponding Measured Responses for Nanoparticle Optimization.

Batch	X_1_: PLGA: NAR Ratio	X_2_: PVA (% *W*/*V*)	Y_1_: Hydrodynamic Diameter (nm)	Y_2_: PDI	Y_3_: EE (%)
PGNP 1	3:1	0.5	289.3 ± 9.4	0.17 ± 0.08	60.89 ± 5.1
PGNP 2	3:1	1.5	307.4 ± 1.4	0.20 ± 0.02	61.34 ± 4.5
PGNP 3	3:1	1	210.7 ± 3.4	0.13 ± 0.02	76.21 ± 2.4
PGNP 4	4:1	0.5	254.2 ± 6.4	0.20 ± 0.03	67.87 ± 4.9
PGNP 5	2:1	1	300.3 ± 10.2	0.14 ± 0.02	64.45 ± 5.6
PGNP 6	3:1	1	209.9 ± 9.2	0.25 ± 0.06	75.42 ± 4.9
PGNP 7	3:1	1	201.2 ± 1.3	0.20 ± 0.07	76.11 ± 2.1
PGNP 8	4:1	1.5	295.4 ± 5.2	0.45 ± 0.06	68.83 ± 3.3
PGNP 9	2:1	0.5	324.9 ± 7.3	0.30 ± 0.08	57.12 ± 4.2
PGNP 10	2:1	1.5	330.4 ± 10.7	0.27 ± 0.03	58.98 ± 4.9
PGNP 11	3:1	1	204.2 ± 4.3	0.11 ± 0.07	75.10 ± 1.2
PGNP 12	3:1	1	203.3 ± 3.2	0.11 ± 0.02	77.23 ± 5.1
PGNP 13	4:1	1	245.2 ± 1.3	0.37 ± 0.07	66.23 ± 1.9

**Table 2:** ANOVA Responses for Optimization of Naringin PLGA NP by Central Composite design.

Analysis of Variance Table [Partial Sum of Squares—Type III] Response Surface 2FI Model												
Source	Sum of Squares			Mean Square			F Value			*p* Value		
	Y_1_ (Hydrodynamic diameter)	Y_2_ (PDI)	Y_3_ (%EE)	Y_1_	Y_2_	Y_3_	Y_1_	Y_2_	Y_3_	Y_1_	Y2	Y_3_
Model	27,221.02	0.09	482.21	5444.20	0.01	96.44	13.11	4.25	4.97	0.001	0.04	0.03
X_1_-PLGA: NAR	4309.44	0.01	83.48	4309.44	0.01	83.48	10.37	3.33	4.30	0.01	0.11	0.07
X_2_-PVA	699.84	0.01	1.78	699.84	0.01	1.78	1.68	2.37	0.09	0.23	0.16	0.77
X_1_X_2_	318.62	0.01	0.20	318.62	0.01	0.20	0.76	4.15	0.01	0.41	0.08	0.92
X_1_^2^	3576.69	0.03	53.56	3576.69	0.03	53.56	8.61	7.06	2.76	0.02	0.03	0.14
X_2_^2^	10,475.52	0.00	205.64	10,475.52	0.003	205.64	25.22	0.83	10.60	0.001	0.39	0.01

**Table 3:** Comparison of PLGA NP and LC PLGA NP for size, PDI and zeta potential.

Parameters	PLGA NP	LC PLGA NP
**Hydrodynamic diameter by DLS**	201.2 ± 1.3 nm	307.6 ± 3.2 nm
**Hydrodynamic diameter using NTA**	196.4 ± 3.8 nm	298.2 ± 5.8 nm
**PDI**	0.20 ± 0.03	0.21 ± 0.05
**Zeta potential**	−19.2 ± 3.4 mV	−28.1 ± 2.9 mV

## Data Availability

All relevant data have been reported in the paper. Additional data used to support the finding are available on request from the corresponding author.

## Abbreviations

The following abbreviations are used in this manuscript:

COPD	Chronic Obstructive Pulmonary Disease
TB	Tuberculosis
PLGA	poly(lactic-co-glycolic acid)
FDA	Food and Drug Administration
NAR	Naringin
SL	Soy lecithin
NP	Nanoparticle
PVA	poly(vinyl alcohol)
LC	Lecithin-coated
LAMA	Long-acting muscarinic acetylcholine antagonists
LABAs	Long-acting β_2_-adrenergic agonists
LPS	Lipopolysaccharides
DPPC	Dipalmitoyl phosphatidylcholine
CCD	Central Composite Design
NTA	Nanoparticle Tracing Analysis
TEM	Transmission Electron Microscopy
FTIR	Fourier-Transform Infrared Red Spectroscopy
DSC	Differential Scanning Calorimetry
XRD	X-ray Diffraction
ROS	Reactive Oxidative stress
IL	Interleukin
YSM	Yolk Sac Membrane
DCFDA	Di-chlorodihydrofluorescein diacetate
SD	Standard deviation
ANOVA	Analysis of variances
MTT	(3-(4,5-Dimethylthiazol-2-yl)-2,5-Diphenyltetrazolium Bromide)
DLS	Dynamic light scattering
